# Gestational weight gain, nutritional status and blood pressure in pregnant women

**DOI:** 10.11606/S1518-8787.2019053000880

**Published:** 2019-07-10

**Authors:** Chiara Alzineth Silva Campos, Maira Barreto Malta, Paulo Augusto Ribeiro Neves, Bárbara Hatzlhoffer Lourenço, Marcia C Castro, Marly Augusto Cardoso

**Affiliations:** I Departamento de Nutrição. Faculdade de Saúde Pública. Universidade de São Paulo, São Paulo, SP, Brasil; II Department of Global Health and Population. Harvard T.H. Chan School of Public Health. Boston, MA, EUA

**Keywords:** Pregnant Women, Weight Gain, Prenatal Nutrition, Maternal and Child Health, Gestantes, Ganho de Peso, Nutrição Pré-Natal, Saúde Materno-Infantil

## Abstract

**OBJECTIVE:**

To evaluate whether weekly gestational weight gain is associated with anemia, vitamin A insufficiency, and blood pressure levels in the third trimester of pregnancy.

**METHODS:**

A prospective study with 457 pregnant women attending primary care in Cruzeiro do Sul, Acre. The weekly gestational weight gain rate measured between the second and third trimesters was classified as insufficient, adequate, and excessive according to the recommendations of the Institute of Medicine 2009. The outcomes at the beginning of the third gestational trimester were: anemia (Hb < 110 g/L), vitamin A insufficiency (serum retinol<1.05 μmol/L) and blood pressure levels (continuous values, in mmHg). Age-adjusted prevalence ratios, schooling, and use of vitamin and mineral supplements were calculated in Poisson regression models with robust variance.

**RESULTS:**

A total of 18.6% of pregnant women had insufficient weekly weight gain, and 59.1% had excessive weight gain. The frequencies of anemia, vitamin A insufficiency and hypertension (systolic blood pressure ≥ 140 mmHg or diastolic ≥ 90 mmHg) were 17.5%, 13.4%, and 0.6%, respectively. The prevalence ratios for anemia among pregnant women with insufficient and excessive weight gain were 0.41 (95%CI 0.18–0.93) and 1.00 (95%CI 0.63–1.59), respectively, when compared to pregnant women with adequate weight gain. For vitamin A insufficiency, the adjusted prevalence ratio was significantly higher among pregnant women with insufficient weight gain (2.85, 95%CI 1.55–5.24) and no difference for excessive weight gain (1.53, 95%CI 0.84–2.74) when compared to pregnant women with adequate weight gain. Pregnant women with excessive weight gain had higher mean systolic blood pressure (111.10; 95%CI 109.9–112.2) when compared to pregnant women with insufficient weight gain (107.50; 95%CI 105.4–109.6) and adequate (106.20; 95%CI 104.3–108.20).

**CONCLUSIONS:**

Insufficient weekly gestational weight gain was associated with the risk of vitamin A insufficiency. Excessive weight gain, in turn, was associated with higher blood pressure values at the beginning of the third gestational trimester.

## RESUMO

**OBJETIVO:**

Avaliar se o ganho de peso gestacional semanal está associado à anemia, à insuficiência de vitamina A e a níveis pressóricos no terceiro trimestre gestacional.

**MÉTODOS:**

Estudo prospectivo com 457 gestantes assistidas na atenção básica em Cruzeiro do Sul, Acre. A taxa de ganho de peso gestacional semanal medida entre o segundo e o terceiro trimestres foi classificada em insuficiente, adequada e excessiva segundo recomendações do *Institute of Medicine* 2009. Os desfechos no início do terceiro trimestre gestacional foram: anemia (Hb < 110 g/L), insuficiência de vitamina A (retinol sérico < 1,05 µmol/L) e níveis pressóricos (valores contínuos, em mmHg). Razões de prevalência ajustadas por idade, escolaridade e uso de suplementos de vitaminas e minerais foram calculadas em modelos de regressão de Poisson com variância robusta.

**RESULTADOS:**

No total, 18,6% das gestantes apresentaram ganho de peso semanal insuficiente e 59,1% ganho de peso excessivo. As frequências de anemia, insuficiência de vitamina A e hipertensão (pressão arterial sistólica ≥ 140 mmHg ou diastólica ≥ 90 mmHg) foram 17,5%, 13,4% e 0,6%, respectivamente. As razões de prevalência para anemia entre gestantes com ganho de peso insuficiente e excessivo foram 0,41 (IC95% 0,18–0,93) e 1,00 (IC95% 0,63–1,59), respectivamente, quando comparadas às gestantes com ganho de peso adequado. Para insuficiência de vitamina A, a razão de prevalência ajustada foi significantemente maior entre gestantes com ganho de peso insuficiente (2,85; IC95% 1,55–5,24) e sem diferença para ganho de peso excessivo (1,53; IC95% 0,84–2,74) quando comparada às gestantes com ganho de peso adequado. As gestantes com ganho de peso excessivo apresentaram valores médios de pressão arterial sistólica maiores (111,10; IC95% 109,9–112,2) quando comparadas às gestantes com ganho de peso insuficiente (107,50; IC95% 105,4–109,6) e adequado (106,20; IC95% 104,3–108,20).

**CONCLUSÕES:**

O ganho de peso gestacional semanal insuficiente foi associado ao risco para insuficiência de vitamina A. O ganho de peso excessivo, por sua vez, foi associado a valores pressóricos maiores no início do terceiro trimestre gestacional.

## INTRODUCTION

Weight gain is an essential factor in the proper course of pregnancy. It is a complex phenomenon, influenced not only by maternal physiological and metabolic changes but also by placental metabolism^1^ . These physiological changes promote the formation and development of amniotic fluid and placenta, increased blood volume, adipose tissue and fetus, and uterine and mammary growth^1^ .

Inadequate pre-gestational or gestational nutritional status and inadequate weight gain during pregnancy are associated with poor reproductive outcomes for both the pregnant woman and the baby^2^ . Low pre-gestational body weight or insufficient gestational weight gain are associated with intrauterine growth retardation, prematurity, and low birth weight of the baby^3^ . On the other hand, pre-gestational obesity or excessive weight gain during pregnancy predisposes women to postpartum hemorrhage, gestational diabetes^4^ , preeclampsia and hypertensive pregnancy^5^ .

Currently, disorders related to increased occurrence of overweight and obesity coexist with micronutrient and vitamin deficiencies in pregnant women^6^ . In this context, anemia stands out as a significant public health problem, currently reaching 29.4% of women in the world, according to estimates for 2011^7^ . Anemia in pregnant women affects the health of both the child and the woman, increasing the risk of low birth weight for the baby^8^ and of death, preeclampsia, cardiovascular alterations, and decreased immunological function for the mother^9^ . Another essential nutrient for women’s health during pregnancy and baby development is vitamin A. During the third gestational trimester, women are more vulnerable to vitamin A deficiency because of physiological increase in blood volume and fetal growth^10^ . With respect to high blood pressure levels, arterial hypertension during pregnancy can lead to metabolic and vascular changes associated with increased maternal cardiovascular risk^11^ . A study conducted in China^5^ showed an association between a higher pre-gestational body mass index (BMI) and excessive gestational weight gain, with a consequent increased risk for hypertensive pregnancy syndrome^5^ .

In Brazil, studies on the relationship between weight gain and nutritional status in pregnancy are scarce, especially in the northern region of the country. According to the latest National Survey of Demographics and Health of Women and Children (PNDS)^12^ , about 29% of women of reproductive age were anemic, and 49% had serum retinol levels below 1.05 μmol/L. Data on the prevalence of anemia and vitamin A status in pregnant women were not collected in the PNDS. Thus, the objective of this study was to evaluate whether weekly gestational weight gain (measured between the second and third gestational trimesters) is associated with the nutritional status of pregnant women at the beginning of the third trimester regarding anemia, vitamin A and blood pressure levels of pregnant women.

## METHODS

A prospective study involving pregnant women, part of the investigation on maternal and child health and nutrition conditions in Acre (MINA-Brazil). Between February 2015 and February 2016, weekly screening of pregnant women enrolled in the Family Health Strategy (FHS) of Cruzeiro do Sul, Acre, was conducted by the team of researchers in partnership with the city’s FHS teams. The inclusion criteria were: gestational age less than 20 weeks based on the date of last menstruation (DLM), having a fixed residence in the municipality, and intention to perform the delivery in the municipality of Cruzeiro do Sul, state of Acre.

The fieldwork team consisted of interviewers (undergraduate nursing and biology students from the Universidade Federal do Acre, Campus Floresta), nursing technicians, nurses, doctors, and researchers (graduate and postdoctoral students) from the Universidade de São Paulo who performed training, supervision and quality control of the data collection. Interviewers conducted home visits to invite participants to the study after clarifying the objectives and stages of the research. After consent, sociodemographic information and reproductive and health history were obtained through a structured questionnaire.

Then, using standardized telephone contact, the first clinical evaluations were performed in the second trimester of pregnancy, based on DLM. During the third gestational trimester, a second evaluation was scheduled based on the estimated gestational age assessed in the first evaluation through obstetric ultrasonography. For the two evaluations done by the research team, data were collected on health conditions and life habits of the pregnant woman, anthropometric data and blood sample for biochemical evaluation.

All anthropometric measurements followed the recommendations of the World Health Organization (WHO)^2^ and were carried out by previously trained staff. The body weight measurement was performed in duplicate with a portable scale from Tanita Corporation, Tokyo (Japan), model UM061, with a capacity of 150 kg and variation of 0.1 kg. Weight was measured with the pregnant woman barefoot, and in light clothes, with upright posture, feet together and arms extended along the body. Height measurement was also performed in duplicate, with bare feet and head free of props and hairstyle, positioned in the center of a portable *Alturaexata stadiometer* , with the precision of 0.1 mm and extension of 213 cm.

The mean weekly gestational weight gain was calculated by the difference in the weight measured between the second and third gestational trimesters, divided by the number of gestational weeks in that period. The weekly weight gain intervals recommended by the Institute of Medicine^1^ were used to classify gestational weight gain as insufficient, adequate and excessive according to pre-gestational self-reported BMI (weight in kg/height^2^ in meters). For pregnant women aged ≥ 19 years, pre-gestational BMI was classified according to WHO criteria^2^: low weight (< 18.5 kg/m^2^ ), adequate (18.5–24.9 kg/m^2^ ), overweight (25.0–29.9 kg/m^2^ ) and obesity (≥ 30 kg/m^2^ ). For pregnant women aged less than 19 years, the pre-gestational nutritional status assessment was performed with the WHO Anthro Plus program^13^ , which calculates BMI and classifies it into z score units for age in adolescents compared to the WHO reference^14^ . We adopted the following as cut-off points: low weight (z score ≤ -2), eutrophic (z score > -2 to z score +1), overweight (z score ≥ +1 to z score +2) and obesity (z score ≥ +2).

Blood pressure measurements were performed at the beginning of the third trimester in duplicates per trained team, using an OMRON HEM-705CPINT digital device. The following recommendations of the Brazilian Ministry of Health^15^ were deemed: after at least five minutes of rest, sitting with the feet on the floor, with the arm at the same level of the heart and use of an arm cuff of appropriate size. Pregnancy arterial hypertension was defined as the presence of systolic blood pressure ≥ 140 mmHg or diastolic blood pressure ≥ 90 mmHg, based on the mean of the two measurements obtained with a 5-minute interval between them^16^ .

Blood samples were obtained by fasting for 8 hours at the beginning of the third trimester. A dry tube (10 mL) for obtaining serum (wrapped with aluminum foil to protect against the light) was kept at room temperature until centrifugation. After centrifugation, the serum was frozen at -20°C and transported within two months to the Human Nutrition Laboratory of the Department of Nutrition, School of Public Health, University of São Paulo, where it was stored at -70°C for further analysis. Blood hemoglobin concentration was analyzed immediately after blood collection with the use of the Hemocue^®^ portable hemoglobinometer (Hb301), using venous blood and cut-off point recommended by WHO^17^: hemoglobin during the first and third gestational trimesters less than 110 g/L at sea level. Serum retinol concentration was evaluated by reverse-phase high-performance liquid chromatography (HPLC)^18^ . According to WHO^19^ criteria, serum retinol levels < 0.7 μmol/L were classified as vitamin A deficiency and 1.05 μmol/L, as vitamin A insufficiency.

For this analysis, the primary exposure variable was the weekly gestational weight gain measured between the second and third gestational trimesters. Outcomes of interest were the presence of anemia and vitamin A insufficiency and variations in blood pressure levels at the beginning of the third gestational trimester. Measurements of central tendency (median, mean and standard deviation) and interquartile ranges (IQ25, IQ75) or 95% confidence intervals (95%CI) were calculated. The chi-square test was used for comparisons between proportions. Multiple Poisson regression models with robust variance were tested for dichotomous outcomes (anemia and vitamin A insufficiency). P values < 0.20 and other theoretical assumptions were adopted for the selection of independent variables for multiple analysis. Data analysis was performed using the statistics package Stata 14.0 while adopting a level of significance of p < 0.05.

The MINA-Brasil study was approved by the Research Ethics Committee of the Faculdade de Saúde Pública of the Universidade de São Paulo (Opinion 872.613, 13 November, 2014). All participants read and signed the informed consent form.

## RESULTS

In total, 860 pregnant women were identified in the FHS prenatal screening in the urban area of Cruzeiro do Sul. Of them, 161 cases were considered ineligible (abortions, more than 20 gestational weeks or residence in another municipality or rural area), and 111 were follow-up losses (refuse to participate, subjects not found, or nonexistent or incomplete contacting information), remaining 588 participants who answered the socioeconomic and health questionnaire. During the follow-up period, 20 (3%) pregnant women were excluded (five abortions, five moved to another municipality and 10 moved to rural areas). There were 40 (7%) losses, including 11 refusals, 17 not found and 12 non-attendances to clinical evaluation, totaling 528 participants (90% of participants) attending the first evaluation. For the second evaluation, 17 (3%) were excluded: four had abortions or stillbirths, three moved to another municipality, six moved to rural areas, and three had twin pregnancies. In addition to these, there were 54 (10%) losses with two refusals, 23 pregnant women who were not found and 29 who did not attend the second evaluation. Thus, a total of 458 (78%) pregnant women performed two clinical evaluations ( Figure ). There were no statistically significant differences between the participants followed in this study and ones without follow-up concerning the following characteristics: age (p = 0.203), skin color (p = 0.127), occupation (p = 0.242), pregnant woman head of household (p = 0.569), schooling (p = 0.052), primigravidae (p = 0.987) and smoking (p = 0.132). The means for schooling (SD) between pregnant women monitored and without follow-up were 10.5 (2.9) and 9.9 (3.1) years of study, respectively.

**Figure:**
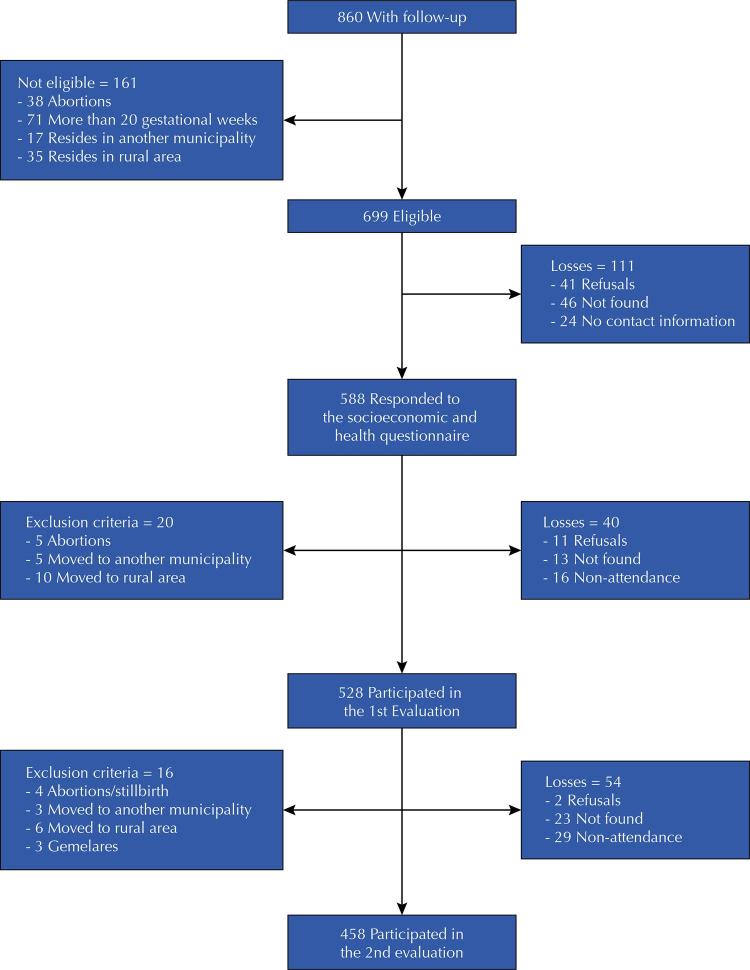
Flowchart of pregnant women with follow-up, eligible for the study, and follow-up losses. Cruzeiro do Sul, state of Acre, 2015–2016.

In general, pregnant women were younger than 30 years (range: 13 to 40 years), with 19% of adolescents (out of 89 adolescents, 16% were between 13 and 16 years old). The majority of the pregnant women self-reported as brown (n = 348, 76%) and living with a partner (n = 360, 79%), 30% had less than nine years of schooling (n = 135) and 39% (n = 179) received social benefits. Less than 50% of the participants were employed, and 14% (n = 62%) were heads of household. As for the number of pregnancies, 44% were primigravidae. The mean gestational age in the second trimester was 19.9 weeks (SD = 2.8), and in the third trimester 27.8 weeks (SD = 1.6).

Table 1 presents the sociodemographic characteristics of the participants according to nutritional status. Age and schooling were positively associated with anemia and not being primigravidae was associated with vitamin A insufficiency. Not having used vitamin supplements during the second or third gestational trimester was associated with anemia and insufficiency of vitamin A.

**Table 1:** Sociodemographic and reproductive characteristics of pregnant women attending prenatal care (n = 458) according to occurrence of anemia and vitamin A insufficiency (VAI). Cruzeiro do Sul, state of Acre, 2015–2016.

Variable	n (%)	Anemia (n, %)	p	VAI (n, %)	p
	
		n = 80		n = 91	
Age (years)					
< 19	89 (19.4)	26 (29.2)	0.001*	23 (25.8)	0.124
≥ 19	369 (80.6)	54 (14.7)		68 (18.6)	
Schooling					
≤ 9 years	135 (29.5)	33 (24.4)	0.011*	24 (18.1)	0.503
> 9 years	323 (70.5)	47 (14.6)		67 (20.8)	
Skin color					
White	70 (15.5)	12 (17.4)	0.978	12 (17.4)	0.556
Non-white	388 (84.5)	68 (17.5)		79 (20.5)	
Marital status					
With partner	360 (78.6)	62 (17.3)	0.800	68 (19.1)	0.332
No partner	98 (21.4)	18 (18.4)		23 (23.5)	
Beneficiary of *Bolsa Família*					
Yes	179 (39.1)	37 (20.7)	0.153*	42 (23.7)	0.113
No	279 (60.9)	43 (15.5)		49 (17.6)	
Primigravida					
Yes	202 (44.1)	37 (18.4)	0.653	48 (23.9)	0.066
No	256 (55.9)	43 (16.8)		43 (16.9)	
Remunerated activity					
Yes	204 (44.5)	30 (14.8)	0.170	39 (19.2)	0.706
No	254 (55.5)	50 (19.7)		52 (20.6)	
Pregnant head of household					
Yes	62 (13.5)	12 (19.7)	0.632	15 (24.2)	0.374
No	396 (86.5)	68 (17.2)		76 (19.3)	
Supplement during 2^nd^ or 3^rd^ trimesters of gestation					
No	227 (49.8)	25 (20.3)	0.040*	35 (28.9)	0.010*
Yes, folic acid or iron, or both	148 (32.3)	40 (20.6)		29 (14.9)	
Yes, multivitamin with vitamin A	82 (17.9)	15 (10.7)		27 (19.4)	

* p < 0.05

Table 2 presents the distribution of weekly gestational weight gain stratified according to BMI categories. Among the pregnant women evaluated, 19% (n = 85) presented insufficient weight gain and 59% (n = 271) presented excessive weight gain. Excessive gestational weight gain was predominant in all pre-gestational BMI categories. The descriptive values of biochemical indicators and pressure levels are presented in Table 3 . High frequencies of anemia (18%), vitamin A deficiency (7%) and insufficiency (13%) were observed at the beginning of the third gestational trimester.

**Table 2:** Classification of weekly gestational weight gain recommended by the *Institute of Medicine* 2009* according to the categories of pre-gestational BMI. Cruzeiro do Sul, state of Acre, 2015–2016. (n = 458)

Pre-gestational BMI Categories	Gestational weight gain		

	Insuficient	Adequate	Excessive
		
	n (%)	n (%)	n (%)
Low weight	11 (33.3)	07 (21.2)	15 (45.5)
Normal	49 (17.3)	70 (24.7)	164 (58.0)
Overweight	17 (15.7)	18 (16.7)	73 (67.6)
Obesity	08 (23.5)	07 (20.6)	19 (55.9)

Total	85 (18.6)	102 (22.3)	271 (59.2)

BMI: body mass index

* Low weight: < 0.44 kg insufficient weight gain; ≥ 0.44 to ≤ 0.58 kg adequate; > 0,58 kg excessive. Normal: < 0.35 kg insufficient weight gain; ≥ 0.36 to ≤ 0.50 kg adequate; > 0,50 kg excessive. Overweight: < 0.23 kg insufficient weight gain; ≥ 0.23 to ≤ 0.33 kg adequate; > 0,33 kg excessive. Obesity: < 0.17 kg insufficient weight gain; ≥ 0.17 to ≤ 0.27 kg adequate; > 0.27 kg excessive.

**Table 3:** Biochemical indicators and blood pressure levels of pregnant women (n = 458) in the third trimester. Cruzeiro do Sul, state of Acre, 2015–2016.

Variable	n	Descriptive Values
Anemia (%, 95%CI)	457	17.5 (14.1–21.3)
Hemoglobin, g/L (mean, SD)	457	119.0 (98.0)
Vitamin A, μmol/L (median, IQ25–75)	455	1.9 (1.2–2.7)
Deficiency of vitamin A, serum retinol < 0.7 μmol/L (%, 95%CI)	455	6.6 (4.5–9.2)
Vitamin A deficiency, serum retinol < 1.05 μmol/L (%, 95%CI)	455	13.4 (10.4–16.8)
Systolic blood pressure, mmHg (mean, SD)	457	109.4 (10.0)
Diastolic blood pressure, mmHg (mean, SD)	457	65.4 (7.5)

SD: standard deviation; IQ: interquartile range

Table 4 presents the prevalence ratios for anemia and vitamin A insufficiency according to the recommendation of weekly gestational weight gain. Insufficient weekly weight gain between the second and third gestational trimesters was associated with a lower frequency of anemia (8%) and higher occurrence of vitamin A insufficiency (33%) compared to pregnant women with adequate weight gain (20% and 12%), respectively (chi-square test, p < 0.05). The prevalence ratios (95%CI) for anemia among pregnant women with insufficient and excessive weight gain were 0.41 (0.19–0.96) and 1.02 (0.64–1.64), respectively, when compared to pregnant women with adequate weight gain (after being adjusted for age, schooling and use of vitamin and mineral supplements). As for vitamin A insufficiency, prevalence among pregnant women with insufficient weight gain (2.88; 95%CI 1.57–5.29) was higher than among those with adequate weight gain (also after adjusting for age, schooling and use of vitamin and mineral supplements). Regarding systolic blood pressure, pregnant women with excessive weight gain had higher mean systolic blood pressure (111.10; 95%CI 109.9–112.2) when compared to pregnant women with insufficient weight gain (107.50; 95%CI 105.4–109.6) and adequate (106.20; 95%CI 104.3–108.20).

**Table 4:** Prevalence, prevalence ratio (PR) and 95% CI for anemia and vitamin A insufficiency according to the classification of weekly gestational weight gain. Cruzeiro do Sul, state of Acre, 2015–2016.

Weekly gestational weight gain	Nutritional indicators in the third trimester of pregnancy					

	Anemia (n = 457)		Vitamin A insufficiency (n = 455)		Systolic blood pressure (n = 457)	Diastolic blood pressure (n = 457)
			
	n (%)	PR (95%CI)*	n (%)	PR (95%CI)*	Mean (95%CI)	Mean (95%CI)
Adequate (n = 102)	20 (19.6)	1	12 (11.8)	1	106.3 (104.4–108.3)	63.9 (62.4–65.4)
Insufficient (n = 85)	7 (8.2)	0.43 (0.19–0.96)	28 (32.9)	2.88 (1,57–5,29)	107.5 (105.4–109.6)	65.1 (63.3–67.0)
Excessive (n = 270)	53 (19.6)	1.02 (0.64–1.60)	51 (19.0)	1.55 (0.86–2.78)	111.1 (109.9–112.3)	66.1 (65.2–66.9)

* PR estimated by Poisson regression model, adjusted for age, schooling and use of nutritional supplements.

Stratified analyses by age group for the association between gestational weight gain, risk of anemia and vitamin A insufficiency showed similar results. Both with adolescent (n = 89) and adult pregnant women (n = 368), anemia was associated with insufficient gestational weight gain (PR = 0.79, 95%CI 0.23–2.37 and PR = 0.32, 95%CI 0.11–0.93, respectively) and excessive (PR = 0.95, 95%CI 0.44–2.04 and PR = 1.05, 95%CI 0.60–1.86, respectively). Similarly, with both adolescent (n = 89) and adult (n = 366) pregnant women, vitamin A insufficiency was associated with insufficient gestational weight gain (PR = 2.98, 95%CI 1.10–8,6 and RP = 2.93, 95%CI 1.38–6.21, respectively) and excessive (PR = 0.94 (95%CI 0.34–2.61 and RP = 1.80; 95%CI 0.88–3.69, respectively). Only the risk for anemia among adolescent pregnant women with insufficient weight gain lost statistical significance (data not shown in the table).

## DISCUSSION

This is the first longitudinal study in a municipality in the interior of Acre, in the Brazilian Western Amazon, to evaluate the association between gestational weight gain, nutritional status, and pressure levels during the third trimester of gestation. Inadequate weekly gestational weight gain was associated with anemia and insufficient vitamin A (insufficient weight gain) and high blood pressure (excessive weight gain) during the third trimester of pregnancy.

The high percentage of inadequate weight gain in our study was also described in previous national studies. The proportion of excessive weight gain in this study was similar to that found by Fernandes et al.^20^ , in a study conducted in Rio de Janeiro, where 66% of overweight pregnant women had excessive weight gain at the end of gestation. Carvalhaes et al.^21^ , in the countryside of São Paulo state, found 78% of excessive gestational weight gain among pregestational overweight women. However, in these studies, there was no investigation on the association between weight gain and the nutritional state of pregnant women.

Comparing our results with the previous studies, we found that even though Cruzeiro do Sul was a municipality with lower human development index (HDI = 0.64)^22^ compared to municipalities in the Southeast region, there was a high frequency of excessive gestational weight gain among pregnant women with pre-gestational overweight. One possible explanation may be the change in the food pattern of the Brazilian population, with a reduction in the intake of minimally processed foods and an increase in the consumption of sugar, fat and ultra-processed foods^23^ . In this case, it is worth emphasizing the importance of prenatal care in the support and protection of pregnant women against common complications of excessive gestational weight gain for both mother and child, such as macrosomia, hemorrhage, and hypertensive gestational disease, contributing to a better indication for childbirth cesarean section^4 , 24^ .

There is a shortage of studies evaluating gestational weight gain per trimester^1^ and the impacts of inadequate weight gain on maternal health. In a multicenter study conducted in Brazil with 2,244 pregnant women, Drehmer et al.^25^ investigated the association between weekly weight gain during the second and third trimesters, ranked according to recommendations from the 2009 Institute of Medicine, and maternal and fetal outcomes. In that study^25^ , in the third trimester, excessive weight gain was associated with preterm birth (RR = 1.70, 95%CI 1.08–2.70) and cesarean delivery (RR = 1.21, 95%CI 1.03–1.44). Also, women with a gestational weight gain lower than recommended for the second trimester had a lower risk for cesarean delivery (RR = 0.82, 95%CI 0.71–0.96) than women with adequate gestational weight gain.

The identification of inadequate weight gain outcomes on maternal nutritional status may be useful for the development of preventive strategies. In this study, the frequency of anemia among women with insufficient weight gain was statistically lower than those with adequate or excessive weight gain. According to a stratified analysis for adolescents and adults, this association remained statistically significant only among adult pregnant women, probably due to the higher number of adult pregnant women. It can be inferred that what occurred, in this case, was hemodilution, a physiological phenomenon in gestation which occurs the increasing in plasma volume higher than the increase in erythrocyte mass, causing a decrease in hemoglobin and hematocrit concentrations, leading to “physiological anemia”^26 , 27^ . Additionally, the pregnant women are more likely to have some regional specificity not identified in this study that contributes to a lower occurrence of anemia at the beginning of the third trimester of gestation, even in the face of a deficit in weight gain.

About the nutritional status of vitamin A, hypovitaminosis A has been considered a public health problem in developing countries. However, there are few studies on the nutritional status of vitamin A in pregnant women in the northern region of Brazil. In our study, when considering all pregnant women, insufficient or excessive gestational weight gain presented a higher chance of vitamin A insufficiency. According to stratified analysis, both adolescent and adult pregnant women with insufficient gestational weight gain presented the risk of vitamin A insufficiency than pregnant women in the same age group with adequate gestational weight gain. This is an important finding that suggests inadequacy in the dietary pattern of these pregnant women, not only concerning to energy intake but also regarding the micronutrient adequacy of the diet. It is likely that the consumption of regional foods naturally rich in beta-carotene (pro-vitamin A), such as pupunha and buriti, among others, is inadequate to our study population, as observed for the Brazilian population in the Pesquisa de Orçamentos Familiares^28^ , which showed a 69% inadequacy in vitamin A intake among women of reproductive age in 2011.

In another study, Dos Santos et al.^29^ compared food intake and prevalence of inadequate nutrient intake among 322 pregnant women, 751 infants, and 6,837 non-pregnant and non-lactating women in a country-level representative sample. In that study, inadequate intake of vitamin A was frequent in non-pregnant and pregnant women, increasing during lactation. In a population-based prospective cohort study with 6,959 mothers and their children in the Netherlands, Gaillard et al. ^30^ evaluated the risk factors for maternal obesity and excessive gestational weight gain and maternal, fetal and infant outcomes. Maternal obesity was associated with increased risk for gestational hypertension (OR = 6.31, 95%CI 4.30–9.26), pre-eclampsia (OR = 3.61, 95%CI 2.04–6.39) and gestational diabetes (OR = 6.28; 95%CI 3.01–13.06).

In this study, excessive gestational weight gain was associated with higher mean systolic blood pressure values. The mean systolic (SBP) and diastolic (DBP) blood pressure observed among participants were 109.4 mmHg and 65.4 mmHg, respectively, considered within average-range values by the Brazilian Society of Cardiology^16^ , which is probably due to the profile of pregnant women enrolled in low-risk prenatal care in primary health care. The occurrence of hypertension in our study was low (three cases, 0.6%). However, pregnant women with excessive gestational weight gain had a higher mean SBP value, which reinforces the need for improvements in maternal care for excessive weight gain during pregnancy, seeking to prevent elevated blood pressure at the end of pregnancy.

Among the limitations of this study, the lack of detailed information on dietary intake during gestation, which was also lacking in previous studies, did not allow to speculate the relationship between insufficient gestational weight gain and bioavailable iron intake for these women. Another significant limitation is related to self-reported pre-gestational weight information, which is susceptible to information bias. However, this limitation was also identified in previous prospective studies because of the difficulty in accurately obtaining this information before gestation. In addition, the location difficulties in our study (due to changes or loss of contact) of the participants contributed to the losses of follow-up between the first (n = 528) and the second evaluations (n = 458). However, considering the nature of prospective studies in Amazonian municipalities, this follow-up loss of less than 20% was similar to that observed in previous epidemiological studies^11 , 20 , 21 , 23^ .

Lastly, among pregnant women enrolled in the FHS prenatal care in the interior of Acre, insufficient gestational weight gain was associated with a lower occurrence of anemia and a higher risk for vitamin A insufficiency; excessive gestational weight gain, in turn, was associated with higher systolic blood pressure values. The findings of this study contribute to the knowledge about the relationship between gestational weight gain and the nutritional status of pregnant women in primary health care. Nutrition care actions related to the promotion of healthy eating practices should be included in the routine of prenatal care to prevent unfavorable outcomes in this population.
